# Supplementary material for: Hub stability in the calcium calmodulin-dependent protein kinase II
Source: Commun Biol. 2024 Jun 25;7:766. doi: 10.1038/s42003-024-06423-y (PMC11199487; doi:10.1038/s42003-024-06423-y)
Supplement: Supplementary file 2 — Supplementary Information [file 42003_2024_6423_MOESM2_ESM.pdf]

SUPPLEMENTARY INFORMATION

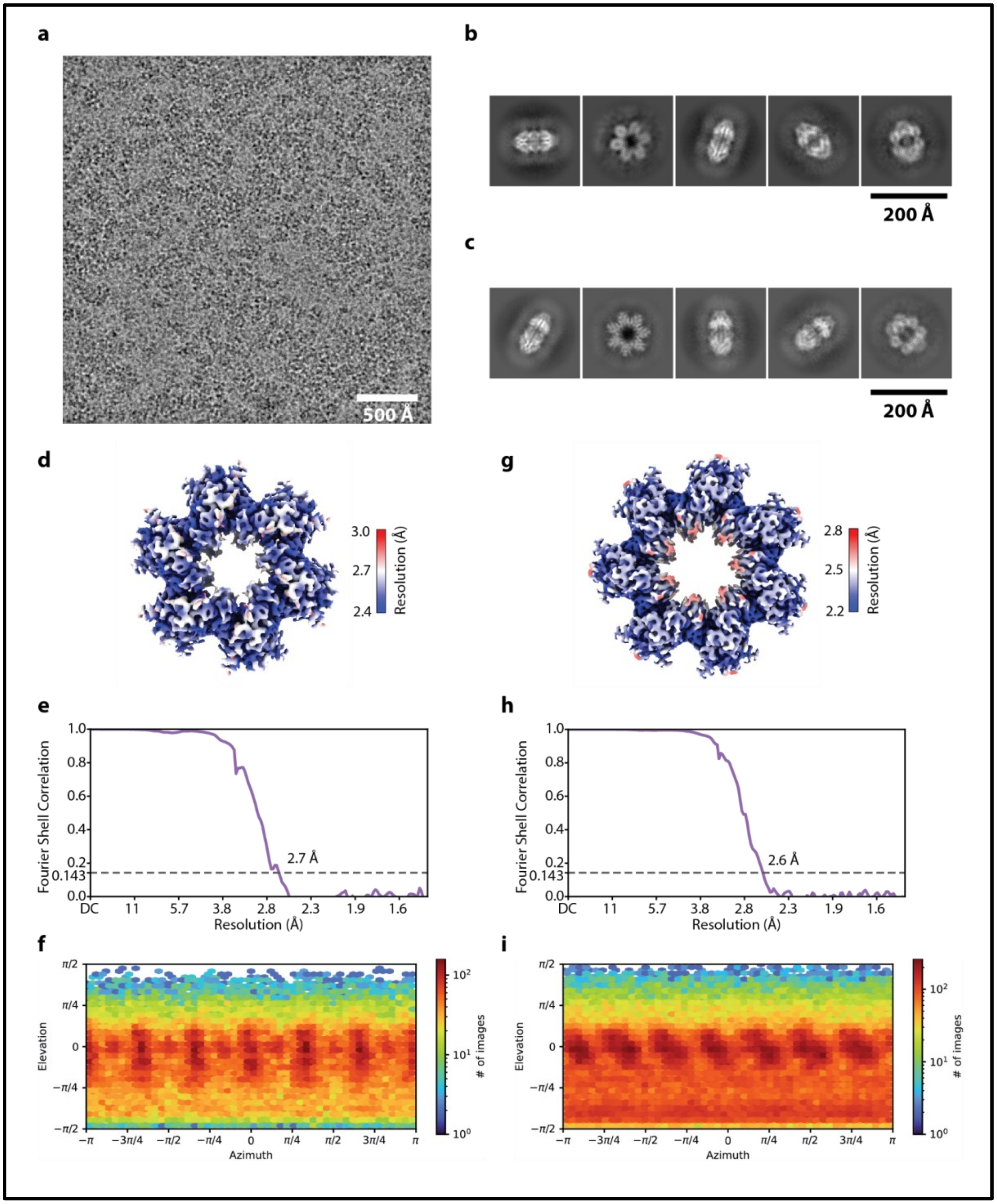

Supplementary Fig 1. Cryo-EM data analysis of CaMKIIα hubs. a. Representative micrograph of CaMKIIα hubs. **b.** Representative particle 2D-class averages**.** CaMKIIα 12-mer hub reconstruction and **c.** CaMKIIα hub 14-mer reconstruction. Local resolution estimation, using CryoSPARC locres function, of **d.** CaMKIIα 12-mer hub, and **g.** CaMKIIα 14-mer hub. Global FSC curve of **e.** CaMKIIα 12-mer hub, and **h.** CaMKIIα 14-mer hub. The resolution as reported by the gold standard FSC (= 0.143, gray dashed line) is 2.7 Å and 2.6

Å, respectively. Angular distribution of the particles used in the final reconstruction of **f.** CaMKIIα 12-mer hub, and **i.** CaMKIIα 14-mer hub.

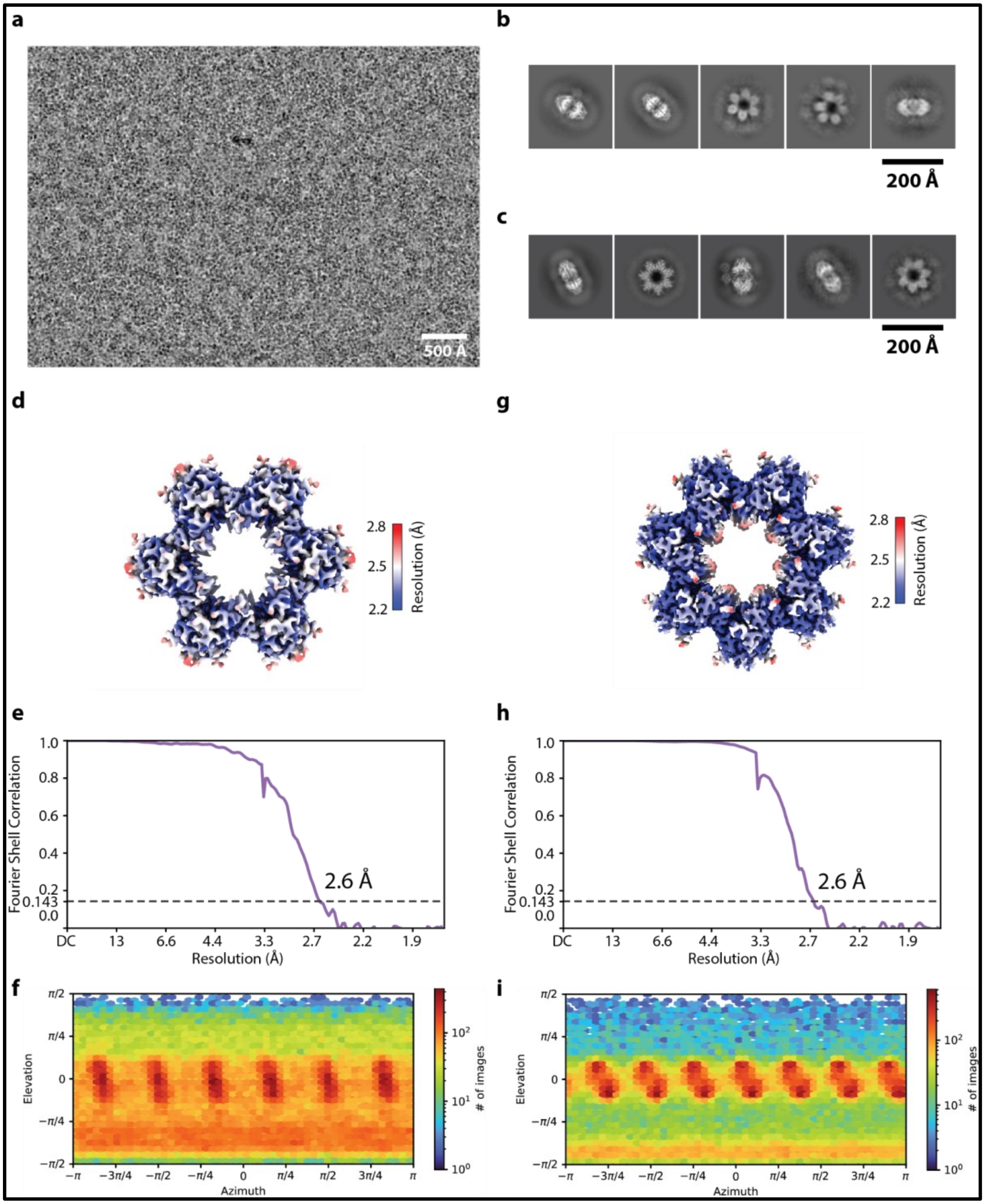

Supplementary Fig 2. Cryo-EM data analysis of CaMKIIβ hubs. a. Representative micrograph of CaMKIIβ hubs. **b.** Representative particle 2D-class averages**.** CaMKIIβ 12-mer hub reconstruction and **c.** CaMKIIβ hub 14-mer reconstruction. Local resolution estimation, using CryoSPARC locres function, of **d.** CaMKIIβ 12-mer hub, and **g.** CaMKIIβ 14-mer hub. Global FSC curve of **e.** CaMKIIβ 12-mer hub, and **h.** CaMKIIβ 14-mer hub. The resolution as reported by the gold standard FSC (= 0.143, gray dashed line) is 2.6 Å and 2.6 Å, respectively. Angular distribution of the particles used in the final reconstruction of **f.** CaMKIIβ 12-mer hub, and **i.** CaMKIIβ 14-mer hub.

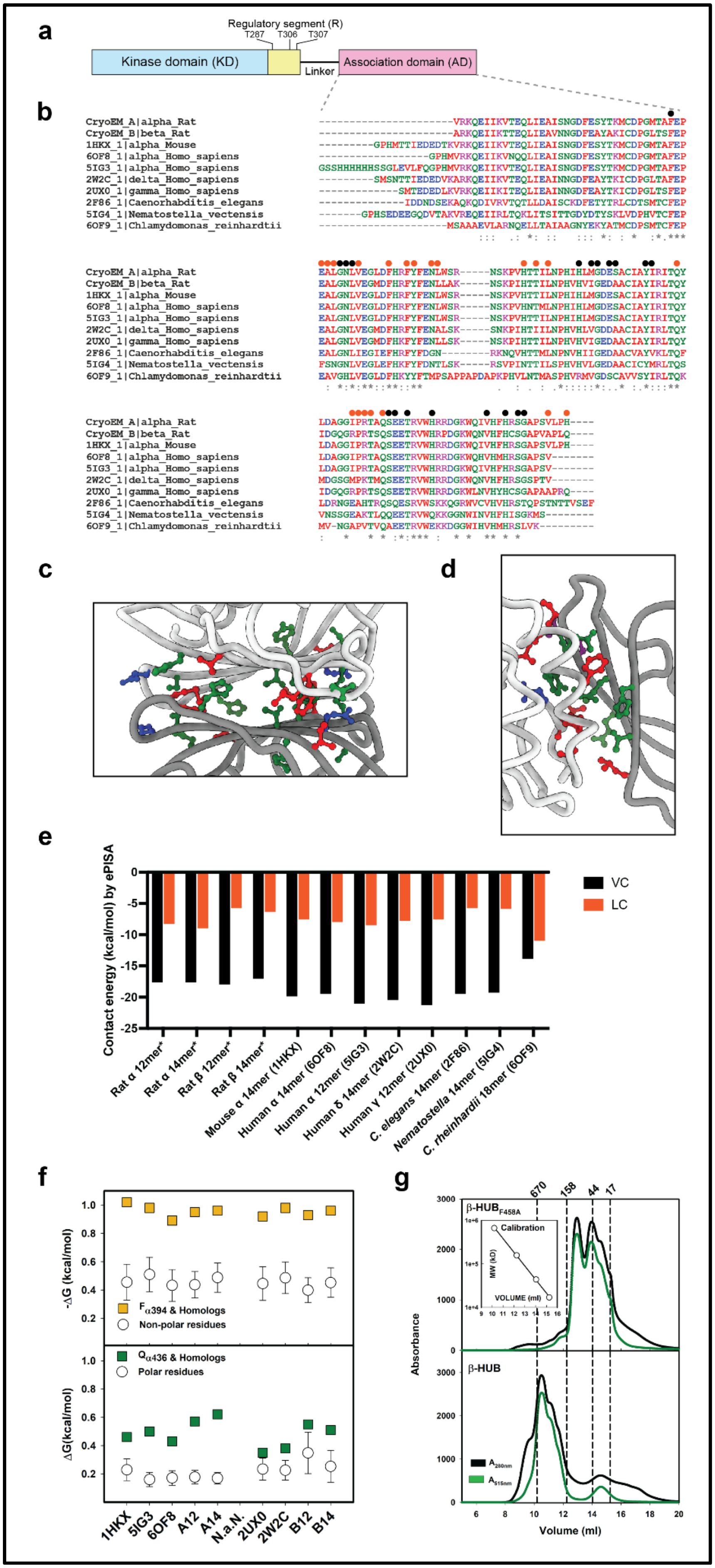
a
Regulatory segment (R)
T287
T306
T307
Kinase domain (KD)
Linker
Association domain (AD)
b
CryoEM_A|alpha_Rat
CryoEM_B|beta_Rat
1HKX_1|alpha_Mouse
6OF8_1|alpha_Homo_sapiens
5IG3_1|alpha_Homo_sapiens
2W2C_1|delta_Homo_sapiens
2UX0_1|gamma_Homo_sapiens
2F86_1|Caenorhabditis_elegans
5IG4_1|Nematostella_vectensis
6OF9_1|Chlamydomonas_reinhardtii
c
d
e
Contact energy (kcal/mol) by ePISA
VC
LC
Rat α 12mer*
Rat α 14mer*
Rat β 12mer*
Rat β 14mer*
Mouse α 14mer (1HKX)
Human α 14mer (6OF8)
Human α 12mer (5IG3)
Human δ 14mer (2W2C)
Human γ 12mer (2UX0)
C. elegans 14mer (2F86)
Nematostella 14mer (5IG4)
C. rheinhardii 18mer (6OF9)
f
-ΔG (kcal/mol)
Fα394 & Homologs
Non-polar residues
Qα436 & Homologs
Polar residues
ΔG(kcal/mol)
1HKX
5IG3
6OF8
A12
A14
N.a.N.
2UX0
2W2C
B12
B14
g
670
158
44
17
β-HUBF458A
Calibration
MW (kD)
VOLUME (ml)
β-HUB
A280nm
A515nm
Absorbance
Volume (ml)

Supplementary Fig. 3: Conserved residues at contact interfaces. a. Diagram of domains of a WT CaMKII holoenzyme. **b.** The multiple sequence alignments of the present and published structure sequences show the conservation of vertical contacts (annotated with black dots) and lateral contacts (annotated with orange dots). Zappo color code identifies residue type. The cartoon representations show the clustering of the residue distribution by type (nonpolar in red, polar in green, acid in purple, basic in blue) at the **c.** vertical and **d.** lateral contact for the α 14-mer hub. **e.** Contact energies (kcal/mol) for VC (black) and LC (orange) estimated by ePISA for high-resolution (< 3.0 Å resolution) hub structures. PDB codes are indicated in the brackets. Structures from this study are indicated by asterisks (*)**. f.** The LC involves 22-24 residues across hubs from mammalian isoforms. The stabilization energy for the burial of the bulky F_{394} residue (yellow squares) is substantially greater than the mean stabilization for other non-polar contact residues. (white circles). The energy cost for the burial of the polar Q_{436} sidechain (green squares), in the absence of hydrogen bond formation, is greater than the mean penalty for other polar contact residues (white circles), **g.** Gel filtration profiles for the β_{F458A} hub and wild type β hub. The N-terminal Venus tag in both constructs (515 nm) juxtaposed with the protein absorbance (380 nm) enabled peak identification. The calibration of the column with MW standards is shown in the top panel (insert).

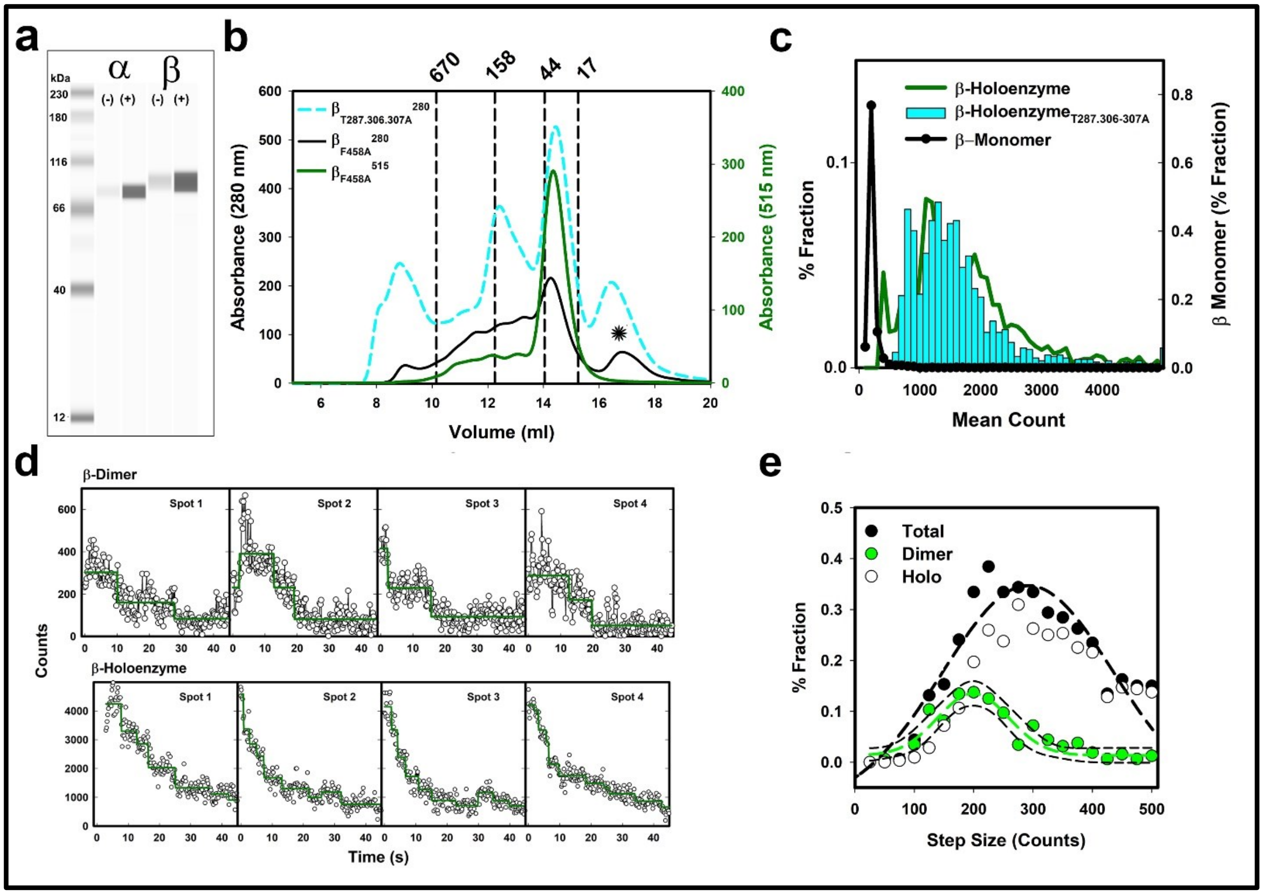

Supplementary Fig. 4: a. Activity in parent Venus-tagged strains with (+) and without calmodulin (-)**. b**. Gel filtration. The β_{F458A} mutation disassembles holoenzymes. The β_{F458A} Venus and protein absorbances are as in Extended Data Fig. 4g. The holoenzyme protein absorbance profile (cyan) has a prominent peak (red asterisk) at the position expected for intact holoenzymes. but peaks at monomer and dimer positions are also prominent. The samples (duplicate runs) were concentrated with 300 kD ultra centrifugal filters in the final purification step; so, the holoenzyme presumably disassembled during the run (1 hour, room temperature). **c-e.** Single-molecule TIRFM**.** Duplicate experiments (> 20 records/experiment), with typically 30->50 immobilized V-CaMKII spots/record were conducted for each condition. Spot intensities were averaged over 20 seconds (100 frames). **c.** The intensity distribution of the monomeric protein was determined in separate experiments. Its mean value ($I_{UNI))}$) was used to estimate assembly size. The native β and $\beta_{T287.306\text{-}307A}$ holoenzyme intensity distributions spanned a broad range with peaks at ($8{*}I_{UNI}$) and ($14{*}I_{UNI}$) respectively and a smaller peak at the dimer ($2{*}I_{UNI}$) modal intensity. **d.** Photobleaching spot records. The β_{F458A} and $\beta_{T287.306\text{-}307A}$ records were filtered to select single dimers ($2{*}I_{UNI}$) or higher order complexes ($>6{*}I_{UNI}$ - $16{*}I_{UNI}<$) respectively. They were fitted with a custom step-finder algorithm to identify stepwise photobleaching events for stoichiometry determination. Top: β_{F458A}. Bottom: β-Holoenzyme. **f.** Photobleaching step-size distributions. The laser excitation and image acquisition were initiated simultaneously for holoenzyme populations, in contrast to dimer populations for which image acquisition was delayed 3-5 seconds after the laser was switched on to allow the autofluorescence to subside. The delay was not feasible for holoenzyme samples since the initial probability for a bleaching event scales with the number of subunits and is, therefore, substantially greater for holoenzyme versus dimer populations. Underweighted subtraction of the autofluorescence combined with double (ΔI =

$2*I_{UNI}$) amplitude steps due to unresolved, closely-spaced, initial bleaching events shifted the holoenzyme distribution to higher values.

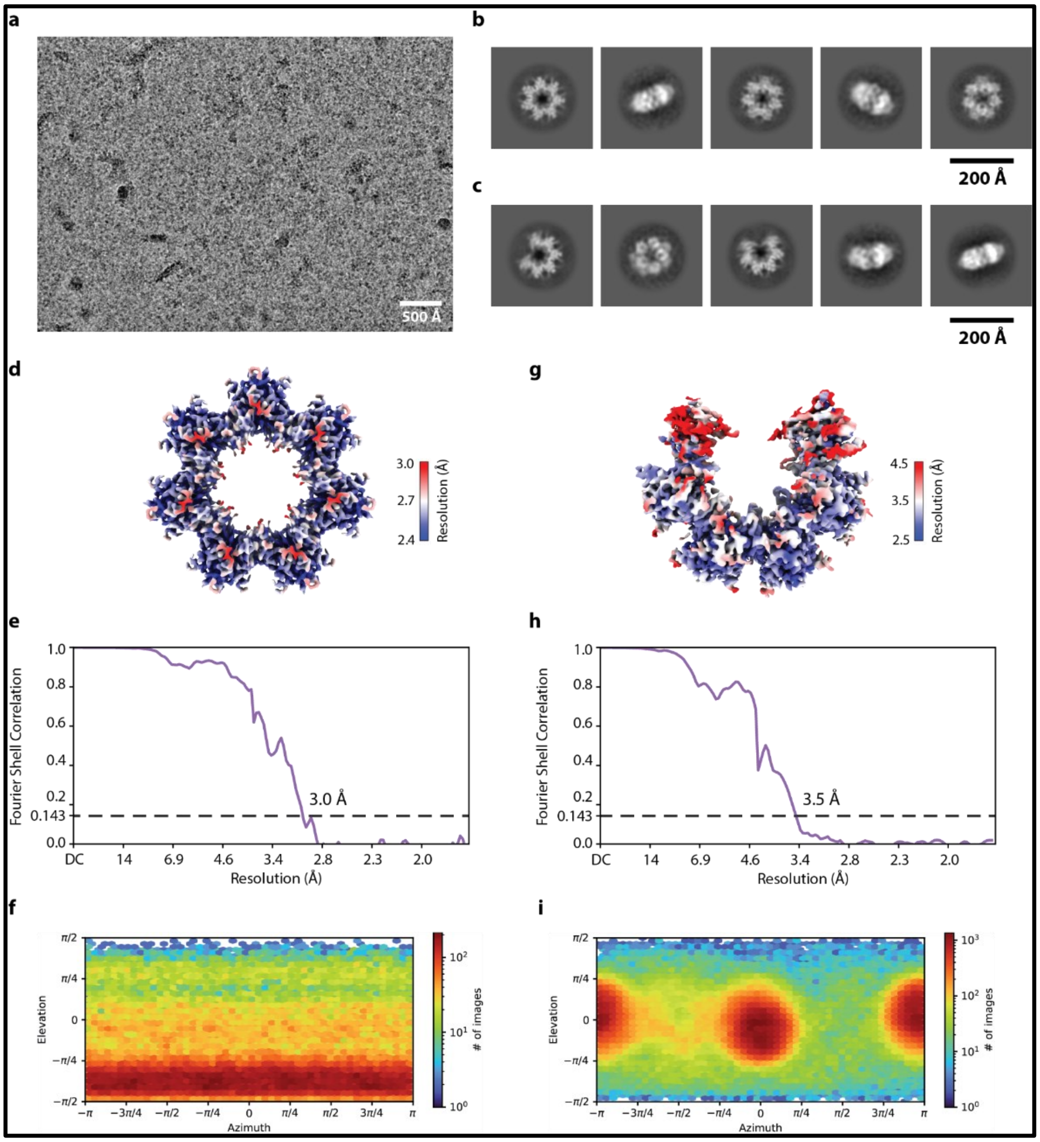

Supplementary Fig 5. Cryo-EM data analysis of CaMKIIβ holoenzyme. a. Representative micrograph of CaMKIIβ holoenzyme. Representative 2D averages of the particles in the final **b.** CaMKIIβ holoenzyme 14-mer reconstruction and **c.** CaMKIIβ holoenzyme 12-mer open ring reconstruction. Local resolution estimation of **d.** CaMKIIβ holoenzyme 14-mer and **g.** CaMKIIβ holoenzyme 12-mer open ring using CryoSPARC *locres* function. Global FSC curve of **e.** CaMKIIβ holoenzyme 14-mer and **h.** CaMKIIβ holoenzyme 12-mer open ring. The grey dashed line indicates the gold standard FSC = 0.143, indicating a resolution of 3.0 Å and 3.5 Å, respectively. Angular distribution of the particles used in the final reconstruction of **f.** CaMKIIβ holoenzyme 14-mer and **i.** CaMKIIβ holoenzyme 12-mer open ring.

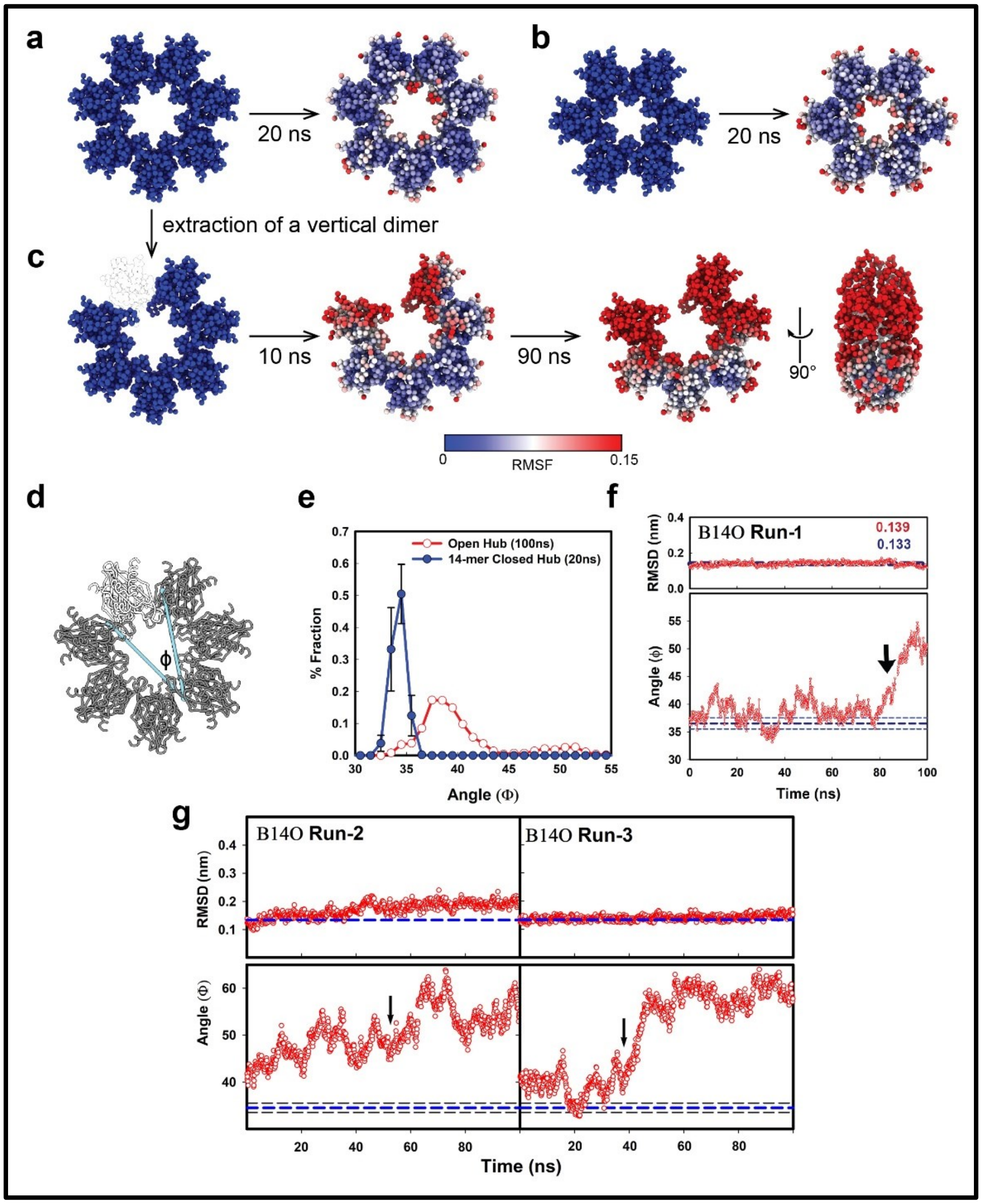

Supplementary Fig. 6: RMSF-derived B-factors calculated from 20 ns MD simulations (3 replicates) of **a.** CaMKIIβ 14-mer hub and **b.** CaMKIIβ 12-mer hub. **c.** The open hub structure constructed from the extraction of a vertical dimer from the 14-mer ring model was simulated for 100 ns (3 replicates). The progressive spread of disorder from the opening with simulation time (run-1) is seen in all replicates. Residues are colored according to the C_α RMSF values (horizontal scale bar). **d.** The missing cone angle, ϕ, is defined as the angle between I498 (chain C), F458 (chain G), and F458 (chain F) (blue). **e.** The increase in angular divergence and spread of the in-silico open ring structure (red (replicate-1)) compared to the distribution for the 14-mer averaged over replicates (blue (mean ± standard error)). **f.** Run-1. Top: The mean RMSD of the C_α atoms of the ADs adjacent to the gap (0.139 nm (red circles)) in the open ring structure compared to the RMSD of the corresponding C_α atoms of the ADs in the closed 14-mer

holoenzyme (0.132 nm (blue (thick line (mean), thin lines (standard error)). The RMSDs were computed relative to the energy-minimized structures. Bottom: The drift in the angle ϕ of the open structure with simulation time (red circles) compared with the mean ϕ (thick dashed line) and $\pm$ σ (thin dashed lines) for the 14-mer holoenzyme. The arrow denotes the episodic increase in ϕ around 85 ns. **g**. Plots for run-2 and run-3, as for run-1 (**f**).

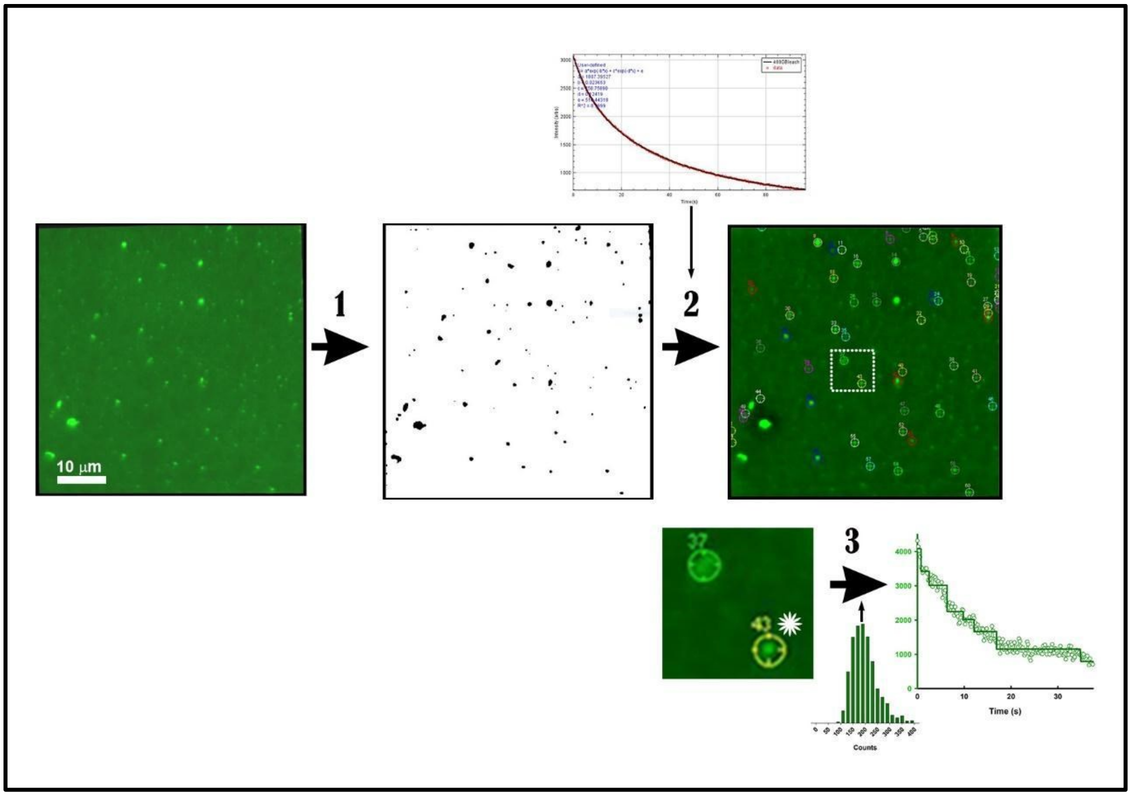

Supplementary Fig. 7: Image processing and photobleaching analysis workflow in the TIRFM experiments. The workflow is illustrated with a video record of a $\beta_{T287.306\text{-}307A}$ holoenzyme sample. Step **(1)** Spot detection. A 100^2-pixel window suppressed spatial inhomogeneity in the critically-illuminated evanescent image field. The top one percentile of spots localized by convolution of a 9x9 pixel LoG with the averaged image stack was marked by the creation of a Boolean mask. Step (**2**) The fluorescence decay of the video record was fitted by a biexponential function for the subtraction of the autofluorescence (decay rate, $k_A = 0.2s^{-1}$) from Venus photobleaching. Locations that did not fit the 5x5 pixel Airy disc point spread function such as small aggregates were rejected as single spots. Step (**3**) Step detection. A custom spot-finder algorithm number and serially processed the spots. The fit to the fluorescence decay from one of the spots (asterisk) shown in the expanded image of the rectangle (white borders) in the numbered image field is shown. The first derivative of the spot intensity was computed over a rolling window of size "**a**" and then smoothed over a sliding window of "**b**" points. Prominent peaks in the output identified abrupt intensity changes using a quality index "**q**" threshold (ImageJ "Analyze Peaks" function); with changes equal to or greater than the mean (I_{UNI}) of the single Venus fluorophore distribution were reported as steps. The detection tolerance is set by the standard deviation of the distribution. Interactive sliders optimized a, b and q slider options for the spot population from a few (<5) spot records.

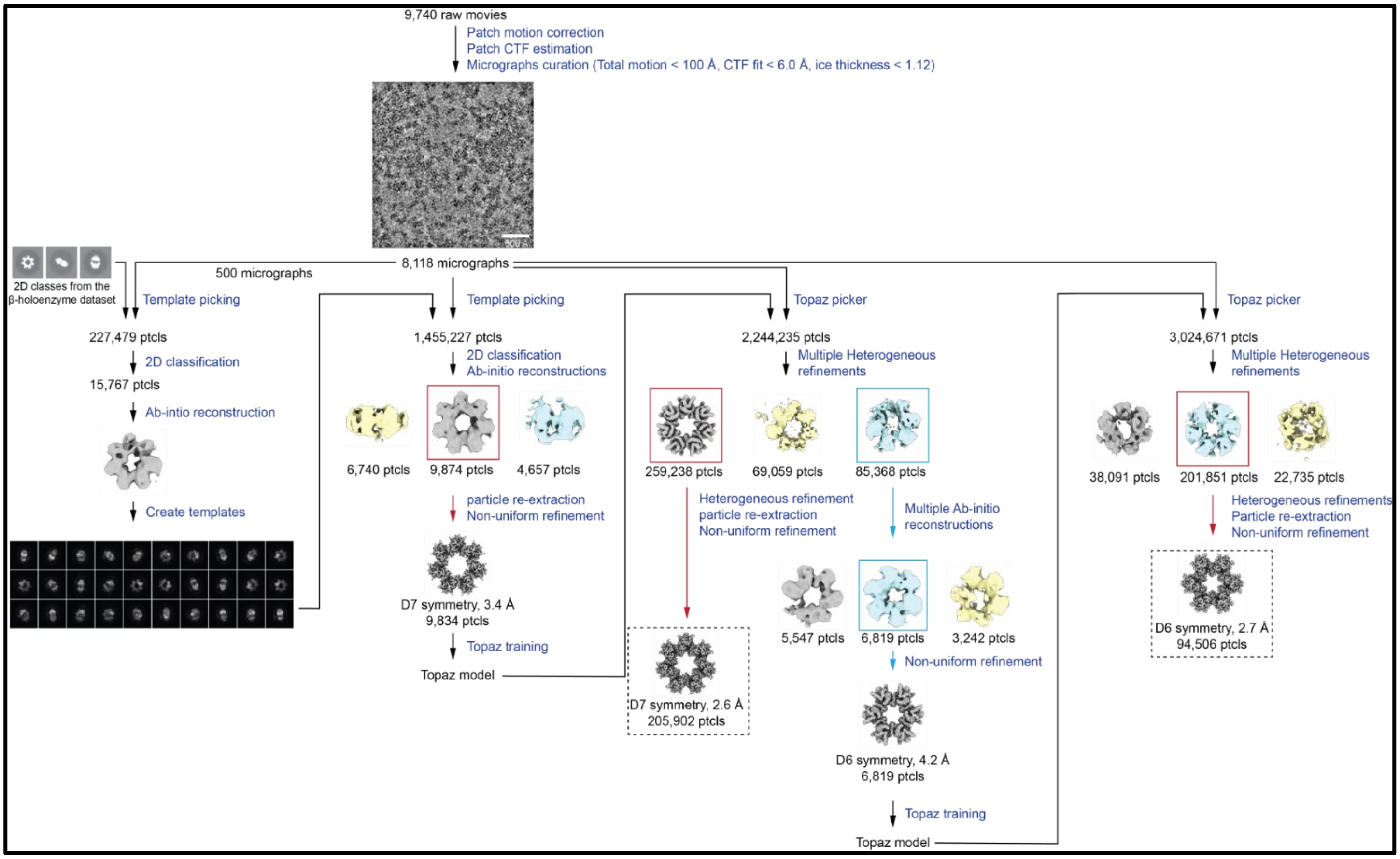

Supplementary Fig 8. Cryo-EM data processing workflow of CaMKIIα hub using CryoSPARC.
9,740 raw movies were pre-processed (motion correction and CTF estimation) and curated in cryoSPARC live. A total of 8,118 micrographs were accepted. The first round of processing used random 500 micrographs for template picking. Three templates from the CaMKIIβ holoenzyme dataset (Extended Data Fig. 10) were low-pass filtered to 20 Å and used to pick 227,479 particles. These particles were pruned using 2D classification followed by an *ab-initio* reconstruction. The map has clear protein features and was used to create 3D templates These templates were used for template picking from the full dataset, 8,118 micrographs, in the second round of processing. The 1,455,227 picked particles were pruned using 2D classifications and *ab-initio* reconstructions, and the final 9,834 particles were reconstructed using non-uniform refinement with D7 symmetry imposed. The map has a 3.4 Å resolution and these particles were considered of good quality. The 9,834 particles were used for Topaz training which picked 2.2 million particles. After several heterogeneous refinements, to clean the dataset, the particles showing D7 symmetrical feature (red box) were selected, re-extracted, and non-uniform refined with D7 symmetry imposed. The final map with 205,902 particles was reconstructed to a 2.6 Å resolution (dotted box). On the other hand, the class showing dodecameric features (cyan box) was further pruned using *ab-initio* reconstructions. The final 6,819 particles were reconstructed to 4.2 Å resolution using a non-uniform refinement with D6 symmetry imposed. These particles were considered of good quality and were used to train a Topaz picker. Around 3 million particles were picked by the Topaz picker followed by several rounds of heterogeneous refinements for selecting particles with D6 symmetrical features. The final map reconstructed from 94,506 particles with D6 symmetry imposed is at 2.7 Å resolution (dotted box).

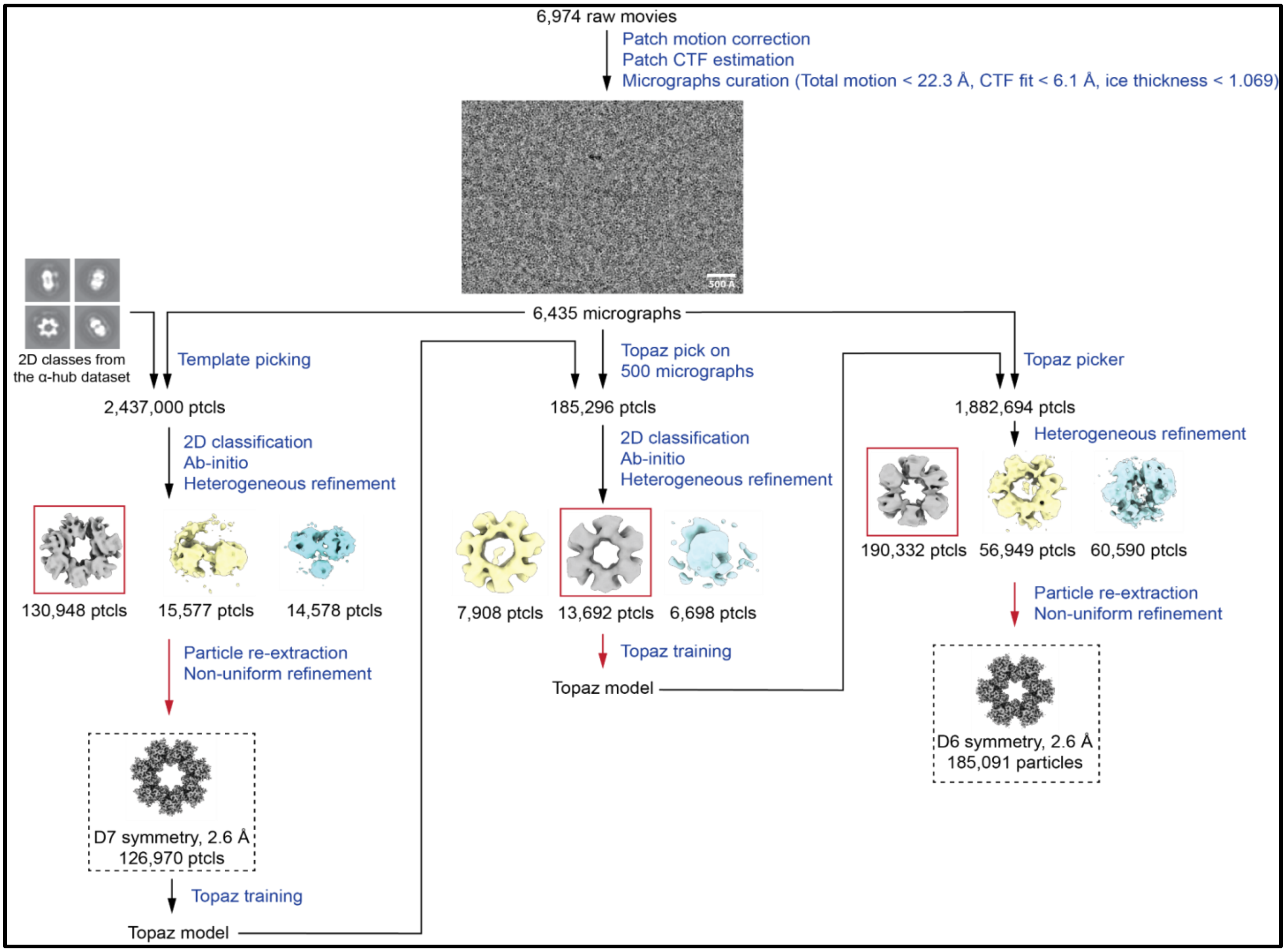

Supplementary Fig 9. Cryo-EM data processing workflow of CaMKIIβ hub using CryoSPARC.
6,974 raw movies were collected and preprocessed (motion correction and CTF estimation) in cryoSPARC live. A total of 6,435 micrographs were accepted after curation. The first round of data processing used template picking on the full 6,435 micrographs. The templates were from the CaMKIIα hub dataset (Extended Data Fig. 8) and low-pass filtered to 20 Å. The template picker picked 2,437,000 particles followed by data pruning using 2D classifications, *ab-initio* reconstructions, and heterogeneous refinements. The class showing clear protein features (red box, 130,948 particles) was selected and re-extracted. The final non-uniform refined map from 126,970 particles with D7 symmetry imposed is at 2.6 Å resolution (dotted box). These particles were used to train a Topaz picker which picked 185,296 particles from random 500 micrographs. The class from heterogeneous refinements showing dodecamer features (red box, 13,692 particles) was selected to train a Topaz picker. The Topaz picker picked 1.8 million particles from the full dataset, 6,435 micrographs. Multiple heterogeneous refinements were used to select particles with D6 symmetrical features. The final 185,091 particles were non-uniform refined to a 2.6 Å resolution map with D6 symmetry imposed (dotted box).

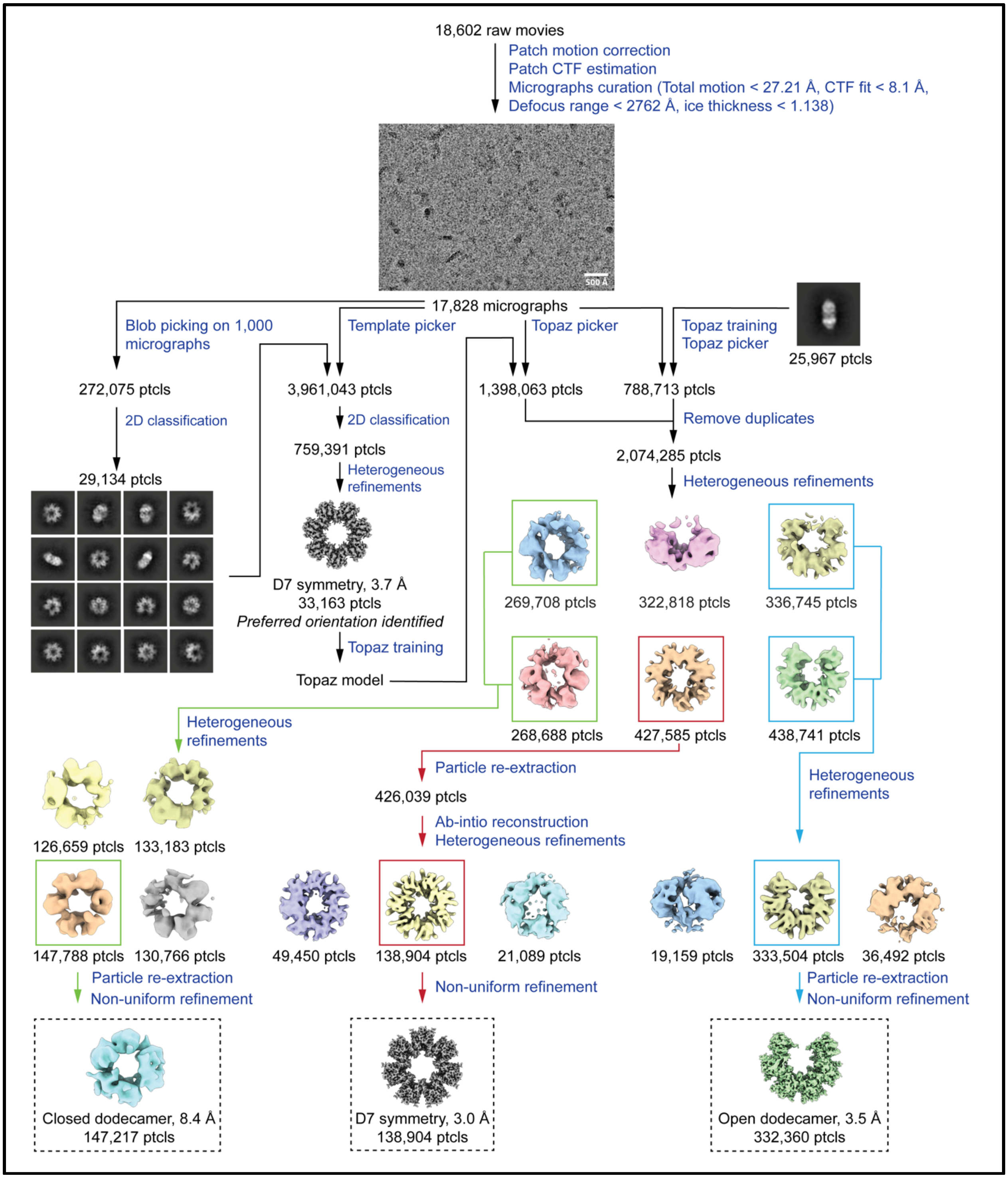

Supplementary Fig 10. Cryo-EM data processing workflow of CaMKIIβ holoenzyme using CryoSPARC. 18,602 raw movies were collected and preprocessed, which includes motion correction and CTF estimation, in cryoSPARC live. A total of 17,828 micrographs were accepted after curation. The first round of processing used blob picking on 1,000 random micrographs followed by two rounds of 2D classifications. The 16 2D averages with clear protein features were used for template picking. The

template picker picked 3,961,043 particles from 17,828 micrographs. After 2D classification and heterogenous refinements, a final map from 33,163 particles was reconstructed to 3.7 Å. These particles were considered of high quality; therefore, they were used to train a Topaz picker, which picked 1.4 million particles. Because the preferred orientation was identified in this 3.7 Å map with streaking artifacts, we trained a separate Topaz picker using 25,967 particles from a side-view 2D averages. This Topaz picker picked 788,713 particles. Two particle stacks picked from the two Topaz pickers were combined, and the duplicates were removed. After heterogeneous refinements, two classes (538,396 particles in total) showed close dodecameric features (green boxes), one class (427,585 particles) showed tetradecameric features (red box), and two classes (775,486 particles in total) showed open dodecameric features (blue boxes). Each of the classes was further pruned using ab-initio reconstructions and heterogeneous refinements for obtaining high-resolution reconstructions. The final three maps (dashed boxes) obtained using non-uniform refinements are an 8.4 Å closed dodecamer (147,217 particles), a 3.0 Å tetradecamer, (D7 symmetry imposed, 138,904 particles) and a 3.5 Å open dodecamer (332,360 particles), respectively.

#	Construct	Primer (Set)	Primer (Name)	Primer (5'-3' Sequence)
1	6His-SUMO-V-rCaMKIIβ*	Vector set:	SATVLBetaIFfor:	CGAGCTCAAGCTTCGAATTCTGCAGTCGACGGTTTAAATGATATCTTTGAAGC
			SATIFrev:	TCCACCAATCTGTTCTCTGTG
		Insert set:	VenforIF:	CCGCGCGGCAGCCATGTGAGCAAGGGCGAGGAGCTG
			VC1LinkIFrev:	CGAAGCTTGAGCTCGAGATC
2	6His-SUMO-V15-mCaMKIIα*	Vector set:	SMT3forIF:	GGATCCACCTCGTCCAATGCAG
			SATIFrev:	TCCACCAATCTGTTCTCTGTG
		Insert set:	ATVC1IFfor:	GAACAGATTGGTGGAGTGAGCAAGGGCGAGGAGC
			VAlpharevIF:	GGACGAGGTGGATCCTCAATGCGGCAGGACGGAG
3	6His-SUMO-V15-rCaMKIIβ**	Deletion set:	VBlinkerModFor:	GCCACCACAGTGACCTGCACC
			VBlinkerModRev:	GTCGACTGCAGAATTCGAAGC
4	6His-V15-rCaMKIIβ*	Deletion set:	VenforIF:	CCGCGCGGCAGCCATGTGAGCAAGGGCGAGGAGCTG
			SMT3revIF:	ATGGCTGCCGCGCGGCACCAG
5	6His-V15-rCaMKIIβ(monomeric/Δ315)**	Deletion set:	rCK2QC315F:	**TGA**TGCCCCGGTGGCCCCGTTACAG
			rCK2QC315R:	**TTA**CACACTGAAATTACGGGTGGC
6	6His-V15-rCaMKIIβ(dimeric/F458A)**	Mutagenesis	ratF458Afor:	**GCC**CACCGTTTCTATTTTGAAAACCTGC
			ratF458Arev:	GTCCATACCCTCCACCAGATTACC
7	6His-V15-mCaMKIIα(dimeric/F394A)**	Mutagenesis	mouseF394Afor:	**GCC**CATCGATTCTATTTTGAAAACTTG
			mouse394Arev:	GTCCAGGCCCTCCACCAGGTTC
8	6His-V15-rCaMKIIβ(T287A)**	Mutagenesis	ratT287Afor:	**GCG**GTGGAATGCCTGAAGAAGTTTAATGC
			ratT287Arev:	CTCCTGGCGATGCATCATGCTTG
9	6His-V15-rCaMKIIβ(T287A/T306A/T307A)**&	Mutagenesis	ratT306/307Afor:	**GCTGCG**ATGCTGGCCACCCGTAATTTCAG
			ratT306/307Arev:	CAGAATGGCGCCCTTTAACTTGC
10	6His-V15-mCaMKIIα-HUB*	Deletion set:	mAhub0IFfor:	AATTCTGCAGTCGACGTGCGCAAACAGGAAATTATCAAAG
			Vck2link4IFrev	GTCGACTGCAGAATTCGAAGC
11	6His-V15-rCaMKIIβ-HUB*	Deletion set:	rBhub0IFfor	AATTCTGCAGTCGACGCCCGCAAGCAGGAAATTATCAAG
			Vck2link4IFrev	GTCGACTGCAGAATTCGAAGC

Supplementary Table 1: Plasmid constructions. *The In-Fusion Snap Assembly cloning system (TaKaRa Bio-USA, San Jose, CA) was used for insertions and some deletions. Larger insertions required amplification of overlapping vectors and insert PCR products as in constructs 1 and 2. Deletions required the generation of a single PCR product with overlapping ends as in construct 4, 11 and 12. **Q5® Site-Directed Mutagenesis Kit (New England Biolabs. Ipswich, MA) was used for all site-directed mutagenesis reactions and the deletion reaction in construct 3. &Mutagenesis was performed sequentially using construct 9 as template for the triple mutant T287.306-307A. The rat, and mouse CaMKII-ADs have 100% sequence identity.

a

NUMBER	SYSTEM	SUBUNITS	PRODUCTION (ns)	BOX SIZE (atoms)	PROTEIN (atoms)	REPLICATES
1	A14LD*	2	40	22882	4278	3
2	A14T*	4	50	44702	8556	3
3	B12T	4	50	41682	8412	3
4	B14T	4	50	43004	8412	3
5	B12Hc	12	20	129453	25217	3
6	B14Hc	14	20	154594	29442	3
7	B14Ho	12	100	123547	25236	4

**Model**	Full-atom	Explicit solvent (TIP3)	**PRE-PRODUCTION**
**Force-field**	CHARMM27		Minimization (400 fr)
**Protonation**	Neutral pH	150 mM NaCl	Equilibration1 (40ps)
**Thermostat**	310°K		Equilibration2 (0.4ns)
**Barostat**	1 atm		
**PME cutoff**	12 angstroms		**VALIDATION METRICS**
**Step**	2 ps/fr		Q-score
*	1 ps/fr		Latent coordinates
**Repository**	Figshare (figshare.com)		
	/s/c59a09308aeebbeda44b		

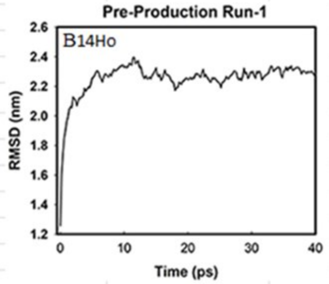

b

STUDY	CITATION	REPOSITORY	OBJECTIVE	METHOD	SYSTEM	STRUCTURE	SPECIES/ISOFORM	DURATION (ns)
1	Stratton_et.al.14	-	Flexibility (rms) analysis of dodecamer contact interfaces	Full atom	Hub	2UXO	Human / "γ"	100
			Dynamics of an open decamer extracted from 12-mer hub	Full atom	Open hub	2UXO	Human / "γ"	100,50
			Docking of R-like peptide into 12-mer hub	Full atom	Vertical Dimer	1HKX	Mouse /"α"	1000
2	Bhattacharya_et.al.16	-	Human versus *S. rosetta* hub β-sheet curvature	Normal Mode	Vertical Dimer	2UXO	Human / "γ"	NA
			Human sequence in *S.rosetta* spiral hub	Full atom	Vertical Dimer	5IGO	Human / "γ"	150,100
3	Karandur_et.al.20	Anton2	CaMKII hub+linker motions	Full atom	Closed Hub	5IG3	Human / "α"	6000
			CaMKII hub+linker+R motions	Full atom	Closed Hub + R	5IG3	Human / "α"	13000
			CaMKII 12-mer hub motions	Normal Mode	Hub	3SOA	Human / "α"	NA
4	Khan.22	Mendeley	Network model of CaMKII 12-mer dynamics	tCONCOORD	Subunit tetramer	3SOA	Human / "α"	NA

Supplementary Table 2: MD Parameters & Citations. a: Top. Table of run duration, box size and replicates. A14LD = Lateral dimer from α 14-mer. A14T = Tetramer from α 14-mer. B12T = Tetramer from β 12-mer. B14T = Tetramer from β 14-mer. B12Hc = Closed β 12-mer hub. B14Hc = Closed β 14-mer hub. B14o = Open β 14-mer diameter hub with 12 ADs. **Bottom (Left -> Right).** System preparation **->** pre-production run durations, validation metrics **->** illustrative RMSD plot for the second B140 replicate (40ps pre-production-1 with initial 400-frame minimization). The structure has relaxed by elimination of steric clashes and stressed bond geometries within 10ps. **b: Publications on hub dynamics.** Method, system and starting structure details for published simulations on the dynamics of CaMKII hubs and sub-assemblies. The full-atom simulations in the first study on the human γ hub are the most related to the present simulations[1]. There are also normal mode simulations of the intrinsic dynamics of the human α hub[2]. The remaining simulations[3,4] examined the interactions of the KD or its R-segment with the human α hub, or the metazoan *S. rosetta* hub

Supplementary References

1 Stratton, M. *et al.* Activation-triggered subunit exchange between CaMKII holoenzymes facilitates the spread of kinase activity. *Elife* **3**, e01610, doi:10.7554/eLife.01610 (2014).

2 Bhattacharyya, M. *et al.* Molecular mechanism of activation-triggered subunit exchange in Ca(2+)/calmodulin-dependent protein kinase II. *Elife* **5**, doi:10.7554/eLife.13405 (2016).

3 Karandur, D. *et al.* Breakage of the oligomeric CaMKII hub by the regulatory segment of the kinase. *Elife* **9**, doi:10.7554/eLife.57784 (2020).

4 Khan, S. Conformational spread drives the evolution of the calcium-calmodulin protein kinase II. *Sci Rep* **12**, 8499, doi:10.1038/s41598-022-12090-y (2022).
