# Supplementary material for: Hub stability in the calcium calmodulin-dependent protein kinase II
Source: Commun Biol. 2024 Jun 25;7:766. doi: 10.1038/s42003-024-06423-y (PMC11199487; doi:10.1038/s42003-024-06423-y)
Supplement: Supplementary file 3 — Description of Additional Supplementary Files [file 42003_2024_6423_MOESM3_ESM.pdf]

Description of Additional Supplementary Files

File name: Supplementary Video 1

Description: **The transition of the CaMKIIβ holoenzyme 14-mer to the 12-mer open hub.** One vertical dimer was removed from the 14-mer then superimposed with the 12-mer open structure using the Matchmaker function (ChimeraX). The movie was produced using the Morph function (ChimeraX) starting from 14-mer to 12-mer open structure. The movie shows the transformation to a slightly open and twisted conformation. The end of the movie shows the Matchmaker superimposition of the subunit from 14-mer and the 12-mer open structures.

File name: Supplementary Video 2

Description: **Flexibility of the CaMKIIβ holoenzyme 12-mer open hub:** 3D-flex analysis for CaMKII β holoenzyme 12-mer open ring was performed using cryoSPARC. Continuous motion along the latent coordinate 1 and 2 was recorded. The movie was made in ChimeraX
